# Effects of Several Tea-like Plants on Liver Injury Induced by Alcohol via Their Antioxidation, Anti-Inflammation, and Regulation of Gut Microbiota

**DOI:** 10.3390/foods13162521

**Published:** 2024-08-13

**Authors:** Jin Cheng, Min Luo, Dan-Dan Zhou, Siyu Huang, Ruogu Xiong, Sixia Wu, Adila Saimaiti, Bangyan Li, Ao Shang, Guo-Yi Tang, Huabin Li

**Affiliations:** 1 Guangdong Provincial Key Laboratory of Food, Nutrition and Health, Department of Nutrition, School of Public Health, Sun Yat-sen University, Guangzhou 510080, Chinasaimaiti@mail2.sysu.edu.cn (A.S.);; 2 Food & Nutritional Sciences Program, School of Life Sciences, Chinese University of Hong Kong, Hong Kong 999077, China; 1155203812@link.cuhk.edu.hk; 3 School of Chinese Medicine, Li Ka Shing Faculty of Medicine, The University of Hong Kong, Hong Kong 999077, China

**Keywords:** alcoholic liver injury, tea-like plant, hepatoprotection, antioxidation, anti-inflammation, gut microbiota

## Abstract

Liver injury induced by alcohol is a serious global health problem. Several tea-like plants are widely used as beverages, which are drunk like tea. In this study, the hepatoprotective effects of eight tea-like plant extracts with the intake of 200 mg/kg.bw/day were investigated and compared using a C57BL/6J mouse model of acute alcohol exposure, including sweet tea, vine tea, *Rabdosia serra* kudo, broadleaf holly leaf, mulberry leaf, bamboo leaf, *Camellia nitidissima*, and *Akebia trifoliata* peels. The results showed that the eight tea-like plants had hepatoprotective effects to different degrees against acute alcohol exposure via enhancing the activities of alcoholic metabolism enzymes, ameliorating oxidative stress and inflammation in the liver, as well as regulating gut microbiota. In particular, sweet tea, bamboo leaf, mulberry leaf, and *Camellia nitidissima* increased the activities of alcohol dehydrogenase or aldehyde dehydrogenase. Among these tea-like plants, sweet tea and *Camellia nitidissima* had the greatest hepatoprotective effects, and their bioactive compounds were determined by high-performance liquid chromatography. Chlorogenic acid, rutin, and ellagic acid were identified in sweet tea, and epicatechin, rutin, and ellagic acid were identified in *Camellia nitidissima*, which could contribute to their hepatoprotective action. These tea-like plants could be drunk or developed into functional food against alcoholic liver injury, especially sweet tea and *Camellia nitidissima*. In the future, the effects of sweet tea and *Camellia nitidissima* on chronic alcoholic liver diseases should be further investigated.

## 1. Introduction

Global alcohol consumption has had an increasing trend in past two decades, was reported to be 6.4 L per capita in 2016, and is forecasted to be 7.6 L in 2030 [1]. The liver is an important organ of ethanol metabolism, where ethanol can be transformed into acetaldehyde by alcohol dehydrogenase (ADH) and further into acetate by aldehyde dehydrogenase (ALDH) [2]. However, highly active acetaldehyde and excessive reactive oxygen species during this process can induce alcoholic liver injury, such as lipid peroxidation, oxidative stress, inflammation, and so on, which can further develop into liver steatosis, liver hepatitis, liver cirrhosis, and liver cancer [2,3]. These alcohol-related liver diseases are a big challenge for public health; for example, alcohol-related deaths from liver cancer in 2019 were estimated to be 91,000, accounting for 20% of global liver cancer deaths [4]. Thus, it is necessary to seek ways to prevent liver injury caused by alcohol. Moreover, the association between gut microbiota and alcohol-related liver disease has been demonstrated by gut–liver axis. Gut microbiota composition can be changed by alcohol, and gut microbiota dysbiosis can induce liver injury [5,6]. For example, endotoxins produced by disorganized microbiota can enter the liver and then directly cause liver injury [7]. Gut dysbiosis can induce disorders of bile acid metabolism and promote liver inflammation, as well as a decrease in bile acid metabolites possessing hepatoprotective effects [8].

Tea-like plants are non-*Camellia sinensis* tea, and their leaves, flowers, rhizomes, skins or branches have been used as beverages, which are widely drunk as tea [9]. They have many health benefits, such as antioxidation, anti-inflammation, and hypoglycemia [9]. Eight commonly used tea-like plants were selected to investigate their protective action against alcoholic liver injury, including sweet tea (ST), vine tea (VT), *Rabdosia serra* kudo (RSK), broadleaf holly leaf (BHL), mulberry leaf (ML), bamboo leaf (BE), *Camellia nitidissima* (CN), and *Akebia trifoliata* peel (ATP). Sweet tea (*Rubus suavissimus*) is a highly sweet natural plant used in medicine and beverage, and has properties of antioxidation, anti-inflammation, and hypoglycemia [10,11]. Vine tea (*Ampelopsis grossedentata*) is widely distributed and used in southern China, and is famous for its antibacterial and antioxidant effects [12]. *R. serra* kudo, belonging to genus *Rabdosia*, is also named “Xi Huang Cao” in Chinese medicine and can ameliorate arthritis [13,14]. Broadleaf holly leaf (*Ilex latifolia* Thunb.), which is also named “KuDing Tea”, is a bitter-tasting herbal tea and is used in Chinese medicine and beverage. It has properties of antioxidation, anti-inflammation, and cancer prevention [15,16]. Moreover, it also reversed the gut microbiota changes induced by a high-fat diet in mice, such as reduced relative abundances of *Erysipelotrichaceae* and *Coriobacteriaceae* [17]. Mulberry is a member of the genus *Morus*, and its leaves have been utilized in medicine and functional food owing to their action of antioxidation and hypoglycemia [18,19]. Bamboo leaf (*Lophatherum gracile*) has abundant flavonoids and possesses activities of antioxidation, obesity prevention, and cardiovascular protection [20,21,22]. *C. nitidissima*, also named as golden-flower tea, has many bioactive compounds, such as flavonoids, polysaccharides, and saponins, and therefore has many health benefits, including antioxidant, antiallergic, and antidepressant effects [23,24,25,26]. *A. trifoliata* is a member of the *Lardizabalaceae* family, and its peels possess the properties of antioxidation, anti-inflammation, and gut microbiota regulation [27,28,29].

The hepatoprotective effects of many plants and dietary components have been reported in the literature, such as green tea, chestnut juice, and ellagic acid [30,31,32,33,34,35]. On the other hand, these tea-like plants are traditionally considered to possess hepatoprotection. However, the hepatoprotective action of these tea-like plants against alcoholic liver injury has not been systematically evaluated and compared in the literature. Thus, the object of this study was to investigate and compare the hepatoprotective effects of eight tea-like plants by a mouse model of acute alcoholic injury. We hope this study provides scientific evidence of the hepatoprotection of these tea-like plants and facilitates their development into functional foods, as well as encourages the public to drink them.

## 2. Materials and Methods

### 2.1. Preparation of Tea-like Plants

The information of eight tea-like plants from China is shown in Table 1. The preparation of tea-like plants was referenced from our previous study [36]. Simulating the conventional brewing of tea, 10 g of each sample and 100 mL boiling distilled water were placed into a round-bottom flask and were extracted in a water bath of 100 °C for 10 min. This extraction of each sample was repeated three times, and extracts were cooled to room temperature and filtered. Afterwards, filtered extracts were concentrated to the volume of 10–15 mL by a rotary evaporator. The concentrated extracts were frozen in a −80 °C freezer for 2 days, then lyophilized, and stored at −20 °C. Before the intervention, the sample extracts were prepared into solution at a concentration of 20 mg/mL (*w*/*v*) using distilled water. In the literature, some studies have used the same weight of raw plant material in consideration of the fact that people usually drink tea infusions with a similar weight or volume in daily life. On the other hand, many studies have used the same weight of the crude extract of plant material [37]. Thus, the same weight of the crude extract of the tea-like plant was used in this study.

### 2.2. Animal and Experimental Design

The C57BL/6J male mice (4–5 weeks old) were bought from the Experimental Animal Center of Guangdong province (Guangzhou, China) and fed in a specific pathogen-free (SPF) animal environment with 40–60% humidity, 22 ± 0.5 °C temperature, and 12 h/12 h light/dark cycle. The Guidelines of the Declaration of Helsinki were followed in this study, and the protocols and procedures were ethically reviewed and approved by the Animal Care Committee at the School of Public Health, Sun Yat-Sen University (No. 2019-002).

The acute alcohol exposure model was performed with reference to a previous study [37], and the dose of tea-like plant extracts was chosen according to the efficient dose of tea extracts detailed in our previous study [36]. Mice were adaptively fed for 1 week, and then randomly divided into 11 groups (n = 8). The groups included control group, model group, positive group, and the tea-like plant intervention groups. The control group underwent intragastrical administration of distilled water at all times. The model group was given intragastrical administration of ethanol firstly, and distilled water was given 30 min after ethanol administration. The intervention groups comprise sweet tea (ST) group, vine tea (VT) group, *Rabdosia serra* kudo (RSK) group, broadleaf holly leaf (BHL) group, mulberry leaf (ML) group, bamboo leaf (BE) group, *Camellia nitidissima* (CN) group, and *Akebia trifoliata* peel (ATP) group. The positive group was given silymarin (SML, a hepatoprotective medicine that is considered a standard). The experiment lasted 15 days. All mice were given free access to water and food. All mice except those in the control group were given 35% (*v*/*v*) ethanol with a dose of 3 g/kg.bw/day intragastrically in the first week and 40% (*v*/*v*) ethanol with a dose of 4 g/kg.bw/day in the second week, and tea-like plant intervention or silymarin of 200 mg/kg.bw/day was given 30 min after ethanol administration. On the last day, 52% (*v*/*v*) ethanol of 5 g/kg.bw/day was given twice by gavage with an interval of 7 h, and intervention or silymarin of 200 mg/kg.bw/day was given 30 min after the first ethanol administration. Two hours after the last ethanol administration, all mice were anesthetized and sacrificed to obtain blood, liver, and fecal samples.

### 2.3. Biochemical Analysis of Serum

After the fresh blood samples were kept at room temperature for 1 h, centrifugation was performed at 2350× *g* for 10 min to attain the serum. Serum alanine aminotransferase (ALT), aspartate aminotransferase (AST), alkaline phosphatase (ALP), triglyceride (TG), and total protein (TP) were measured by automated biochemistry analyzer. The procedures were conducted strictly according to the manufacturer’s instructions. The concentrations of ALT, AST, and ALP were tested by rate method, TG was tested by enzymatic method, and TP was tested by biuret method.

### 2.4. Histopathological Analysis of Liver

Hematoxylin–eosin (H&E) staining was used to analyze the morphology of the livers. The livers were rinsed by pre-cooled PBS solution, cut into a 5 μm thick pieces, and fixed in 4% paraformaldehyde for histopathological analysis. Photos were obtained using a light microscope.

### 2.5. Analysis of Oxidative Stress Indices, TG, ADH, and ALDH in Liver

The 200 mg liver tissue was weighed and mixed with 1.8 mL 0.9% NaCl solution. The mixture was grinded with a tissue grinder (20 times/s; 2 min) to attain a 10% (*w*/*v*) liver tissue homogenate and centrifuged at 1500× *g* for 10 min to obtain supernatant for test. The concentrations of TG, malondialdehyde (MDA), glutathione (GSH), and TP were tested by kits from Nanjing Jiancheng Bioengineering Institute (Nanjing, China). The activities of alcohol dehydrogenase (ADH), aldehyde dehydrogenase (ALDH), superoxide dismutase (SOD), and glutathione peroxidase (GSH-Px) were tested by kits from Nanjing Jiancheng Bioengineering Institute (Nanjing, China). The procedures were strictly performed according to the manufacturer’s instructions.

### 2.6. Analysis of Inflammatory Indices in Liver

The 25 mg liver tissue was weighed, mixed with PBS solution, and grinded to attain a 10% (*w*/*v*) liver tissue homogenate. The supernatant was obtained by centrifugation for test. The concentrations of interleukin (IL)-1β, IL-6, and tumor necrosis factor-α (TNF-α) were measured by kits from Guangzhou Onfei Biotechnology Co., Ltd. (Guangzhou, China). The procedures were strictly performed according to the manufacturer’s instructions.

### 2.7. Composition and Structure Analysis of Gut Microbiota

The fecal samples were collected aseptically after last ethanol gavage, quickly frozen in liquid nitrogen and then stored at −80 °C. The 16S rRNA sequencing technique was used to analyze the composition and structure of gut microbiota. The concentration and purity of DNA were tested by Thermo NanoDrop One (Waltham, MA, USA) after extraction. Using genomic DNA as a template, the 16S rRNA gene in the V3–V4 region was selected and amplified by primers 5′-ACTCCTACGGGAGGCAGCA-3′ and 5′-GGACTACHVGGGTWTCTAAT-3′, and then an amplicon was generated by specific primers with a barcode and TaKaRa Premix Taq^®^ Version 2.0 (Dalian, China). The length and concentration of the PCR amplicon products were detected by 1% agarose gel electrophoresis and those whose bright main strips were within the normal range were used for further experimentation. PCR products were mixed in equidensity ratios after determining the concentrations by GeneTools Analysis Software (Version 4.03.05.0, SynGene), and then the mixture was purified with an E.Z.N.A. Gel Extraction Kit (Omega, Norcross, GA, USA). Sequencing libraries were generated using NEBNext^®^ UltraTM II DNA Library Prep Kit for Illumina^®^ (New England Biolabs, Ipswich, MA, USA), and sequenced on an Illumina Nova6000 platform. Finally, 250 bp paired-end reads were generated. α-diversity analysis was conducted by ACE, Chao1, Simpson, and Shannon indices. β-diversity analysis was performed by principal co-ordinate analysis (PCoA) with a permutational multivariate analysis of variance (PERMANOVA) (Bray–Curtis distance). Microbial community composition was analyzed at the phylum and genus levels. Linear discriminant analysis effect size (LEfSe) was analyzed to determine the biomarkers of each group (LDA score ≥ 2).

### 2.8. Determination of Bioactive Compounds in Tea-like Plants

According to the results, two tea-like plants that had great ameliorative effects against acute alcoholic liver injury were picked out and their bioactive compounds were determined using the high-performance liquid chromatography (HPLC) method, referring to our previous study [27].

### 2.9. Statistical Analysis

All tests were repeated in three times. Data were expressed as mean ± standard deviation (SD) and analyzed by SPSS 20.0 statistical software. Moreover, one-way analysis of variance (ANOVA) followed by least-significant difference (LSD) was used in this study (α = 0.05). GraphPad Prism 8.0 was used to draw the graphs.

## 3. Results

### 3.1. Effects of Tea-like Plants on Alcohol Metabolism

**Figure 1:**
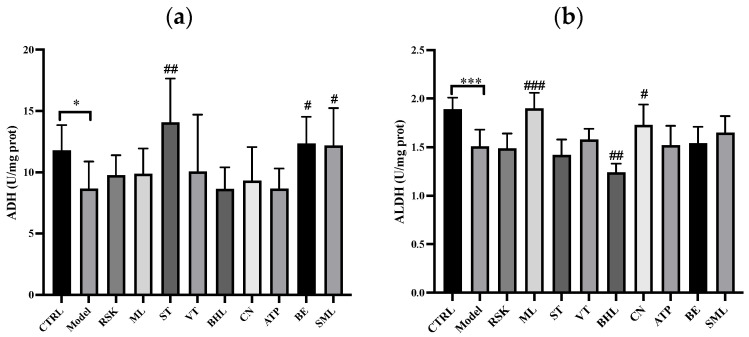
Effects of eight tea-like plants and silymarin on enzymes in alcohol metabolism: (**a**) alcohol dehydrogenase (ADH); (**b**) aldehyde dehydrogenase (ALDH). * *p* < 0.05 and *** *p* < 0.001 in the comparison between control group and model group. # *p* < 0.05, ## *p* < 0.01, and ### *p* < 0.001 in the comparison between intervention group and model group. CTRL: control group; Model: model group; RSK: *Rabdosia serra* kudo group; ML: mulberry leaf group; ST: sweet tea group; VT: vine tea group; BHL: broadleaf holly leaf group; CN: *Camellia nitidissima* group; ATP: *Akebia trifoliata* peel group; BE: bamboo leaf group; SML: silymarin (positive) group.

ADH and ALDH are the main enzymes of alcohol metabolism in the liver [36,38]. As shown in Figure 1, the model group had lower ADH activity (*p* < 0.05) and ALDH activity (*p* < 0.001) in the liver in the comparison with the control group. The inhibition of ADH and ALDH activities by acute alcohol exposure was also reported in a previous study [39]. The lower activities of ADH and ALDH indicated that alcohol metabolism disorders were induced in the model group and especially lower ALDH activity hindered the transformation from acetaldehyde to acetate, leading to strong liver toxicity. Silymarin, which is universally considered to ameliorate liver injury, raised ADH activity significantly (*p* < 0.05), and ALDH activity slightly without significant differences (*p* > 0.05). Compared with the model group, sweet tea (ST) and bamboo leaf (BE) increased ADH activity by 62.04% (*p* < 0.01) and 42.27% (*p* < 0.05), which were both higher than that of silymarin (40.52%). Moreover, *Rabdosia serra* kudo (RSK), vine tea (VT), and mulberry leaf (ML) all had an increasing trend in ADH activity, although there were no significant differences (all *p* > 0.05). Other tea-like plant extracts had no effects on ADH activity (all *p* > 0.05).

Additionally, *Camellia nitidissima* (CN) and mulberry leaf (ML) ameliorated the decrease in ALDH activity caused by alcohol (*p* < 0.05 and *p* < 0.001, respectively), but broadleaf holly leaf (BHL) further inhibited ALDH activity. These results indicated that ML and CN could promote the transformation from acetaldehyde to acetate. However, BHL might deteriorate the accumulation of acetaldehyde. Moreover, *Akebia trifoliata* peel (ATP) and bamboo leaf (BE) had an uptrend towards ALDH activity, but no significant differences were found (both *p* > 0.05). Other tea-like plant extracts had no effects on ALDH activity (all *p* > 0.05).

In summary, ST, BE, ML, and CN reversed alcohol metabolism disorders to some extent, but BHL might make alcohol metabolism disorders worse, which should be avoided in alcohol exposure.

### 3.2. Effects of Tea-like Plants on Liver Function Indices

**Figure 2:**
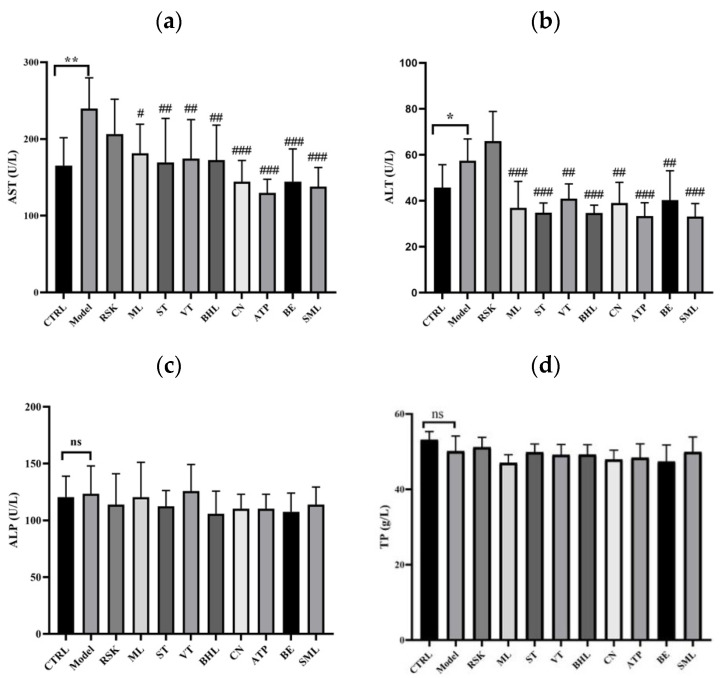
Effects of eight tea-like plants and silymarin on liver function indices: (**a**) serum aspartate aminotransferase (AST); (**b**) serum alanine aminotransferase (ALT); (**c**) serum alkaline phosphatase (ALP); (**d**) serum total protein (TP). * *p* < 0.05 and ** *p* < 0.01 in the comparison between control group and model group. “ns” means there were no significant differences between control group and model group. # *p* < 0.05, ## *p* < 0.01, and ### *p* < 0.001 in the comparison between intervention group and model group. CTRL: control group; Model: model group; RSK: *Rabdosia serra* kudo group; ML: mulberry leaf group; ST: sweet tea group; VT: vine tea group; BHL: broadleaf holly leaf group; CN: *Camellia nitidissima* group; ATP: *Akebia trifoliata* peel group; BE: bamboo leaf group; SML: silymarin (positive) group.

The process of alcohol metabolism can produce acetaldehyde and further form toxic adducts by binding to protein and DNA, finally leading to liver injury [40]. In this study, ALT, AST, ALP, and TP levels were tested to analyze the liver function. As shown in Figure 2a,b, compared with the control group, alcohol increased serum levels of AST and ALT in mice by 44.99% and 25.68%, respectively (both *p* < 0.05), implying that alcohol induced liver damage in the mice. Mulberry leaf (ML), sweet tea (ST), vine tea (VT), *Camellia nitidissima* (CN), broadleaf holly leaf (BHL), *Akebia trifoliata* peel (ATP), bamboo leaf (BE), and silymarin (SML) effectively inhibited the alcohol-induced increase in AST level (all *p* < 0.05) in comparison with the model group. Among them, ATP exhibited the best inhibitory effect on AST level of 45.87%, even better than that of silymarin (42.54%). Moreover, *Rabdosia serra* kudo (RSK) did not influence the level of AST (*p* > 0.05). As for ALT level, mice in the ML, ST, VT, CN, BHL, ATP, BE, and SML groups all had lower levels of ALT than those in the model group (all *p* < 0.01). Moreover, ATP, BHL, and ST extracts had the best inhibitory effects on ALT, with a reduction of 42.03%, 39.71%, and 39.42%, respectively, whereas RSK extract had no effects on ALT (*p* > 0.05). As shown in Figure 2c,d, alcohol, SML, and the eight tea-like plant extracts did not influence the levels of ALP or TP (all *p* > 0.05).

Overall, ML, ST, VT, BHL, CN, ATP, and BE maintained the liver function away from alcohol-induced injury, especially ATP. One previous study reported that *Camellia nitidissima* extract with doses of 40–160 mg/kg.bw/day decreased the levels of AST and ALT in a mouse model of acute liver injury [41], which was consistent with our findings. Moreover, current studies about the health benefits of *Akebia trifoliata* peel mostly focus on antioxidation and anti-inflammation [27,29,42,43]; thus, our finding that *Akebia trifoliata* peels remarkably reduced the levels of ALT and AST could provide a new direction of research, for example, whether ATP ameliorates the disorders of liver function in chronic alcohol liver disease or non-alcoholic fatty liver disease can be further investigated.

### 3.3. Effects on Serum Lipid and Hepatic Lipid Indices

**Figure 3:**
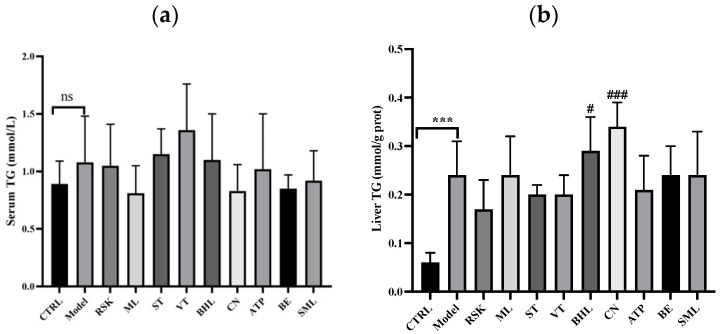
Effects of eight tea-like plants and silymarin on lipid indices: (**a**) serum triglyceride (TG); (**b**) liver TG. *** *p* < 0.001 in the comparison between control group and model group. “ns” means there were no significant differences between control group and model group. # *p* < 0.05 and ### *p* < 0.001 in the comparison between intervention group and model group. CTRL: control group; Model: model group; RSK: *Rabdosia serra* kudo group; ML: mulberry leaf group; ST: sweet tea group; VT: vine tea group; BHL: broadleaf holly leaf group; CN: *Camellia nitidissima* group; ATP: *Akebia trifoliata* peels group; BE: bamboo leaf group; SML: silymarin (positive) group.

Acute alcohol exposure might induce lipid metabolism dysfunction; therefore, serum and hepatic TG levels were tested. As shown in Figure 3a, acute alcohol exposure did not have any impact on serum TG, nor did SML or the eight tea-like plant extracts (all *p* > 0.05). As shown in Figure 3b, liver TG level was significantly increased in the model group, compared with that in the control group (*p* < 0.001). *Rabdosia serra* kudo (RSK), sweet tea (ST), vine tea (VT), *Akebia trifoliata* peel (ATP), and bamboo leaf (BE) extracts reversed liver TG level slightly, though there were no significant differences when compared with model group (all *p* > 0.05), whereas broadleaf holly leaf (BHL) and *Camellia nitidissima* (CN) extracts further increased liver TG level.

Alcohol can induce lipid accumulation in the liver by influencing the lipid uptake, de novo lipid synthesis, fatty acid oxidation, lipid export, and lipid droplet formation and catabolism in the liver [44]. In our study, liver TG level was elevated by alcohol, indicating that lipid metabolism disorders in the liver may have occurred. However, none of the tea-like plant extracts decreased the liver TG level in the intervention groups compared with the model group, and even the liver TG levels in the CN and BHL groups were higher than in the model group. The result that CN did not ameliorate lipid index at a dose of 100 mg/kg.bw/day has been reported before [45].

### 3.4. Hepatic Histopathological Analysis

**Figure 4:**
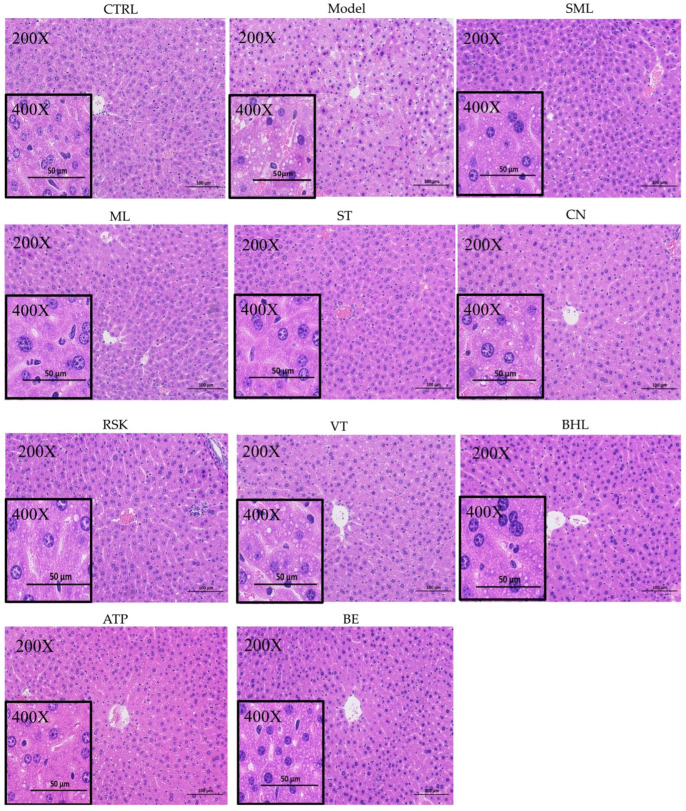
H&E staining results of livers in mice (magnification: 200 or 400). CTRL: control group; Model: model group; SML: silymarin (positive) group; ML: mulberry leaf group; ST: sweet tea group; CN: *Camellia nitidissima* group; RSK: *Rabdosia serra* kudo group; VT: vine tea group; BHL: broadleaf holly leaf group; ATP: *Akebia trifoliata* peel group; BE: bamboo leaf group.

The effects of alcohol, silymarin, and the eight tea-like plant extracts on the liver were further assessed by H&E staining. As shown in Figure 4, the liver in the control group displayed a regular arrangement of hepatic cells. However, lipid droplets and disordered arrangement of hepatic cells occurred in the livers of mice after the 15-day exposure to alcohol, but silymarin (SML) reversed hepatic abnormalities. The eight tea-like plant extracts alleviated alcohol-induced abnormalities in the liver to varying degrees. The livers in the tea-like plant extract intervention groups except the RSK group showed a relatively regular arrangement of hepatic cells. In particular, ML and ST significantly reduced the lipid droplets in the livers of mice, and CN, VT, BHL, ATP, and BE alleviated the formation of lipid droplets slightly.

### 3.5. The Effects of Tea-like Plants on Oxidative Stress in the Liver

**Figure 5:**
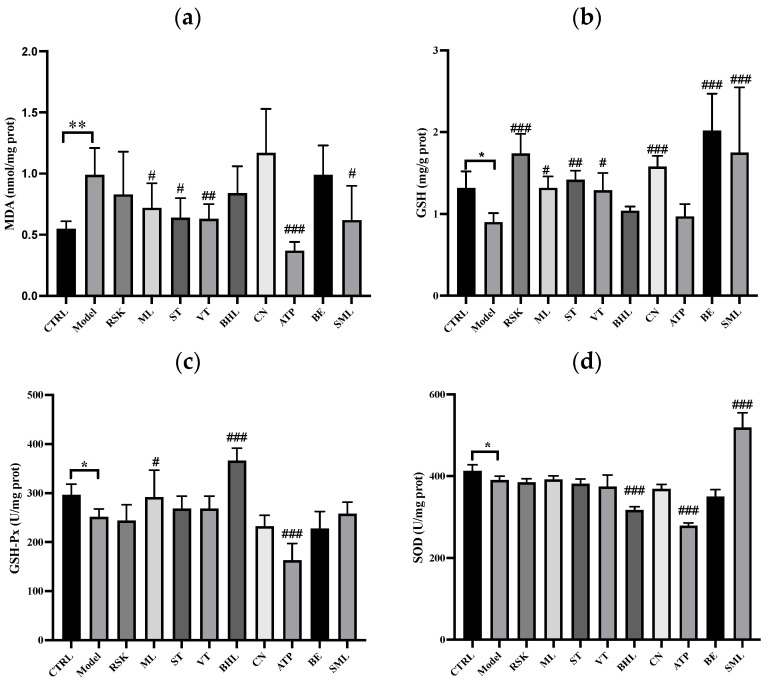
Effects of eight tea-like plants and silymarin on oxidative stress in the liver: (**a**) hepatic malondialdehyde (MDA); (**b**) hepatic glutathione (GSH); (**c**) hepatic glutathione peroxidase (GSH-Px); (**d**) hepatic superoxide dismutase (SOD). * *p* < 0.05 and ** *p* < 0.01 in the comparison between control group and model group. # *p* < 0.05, ## *p* < 0.01 and ### *p* < 0.001 in the comparison between intervention group and model group. CTRL: control group; Model: model group; RSK: *Rabdosia serra* kudo group; ML: mulberry leaf group; ST: sweet tea group; VT: vine tea group; BHL: broadleaf holly leaf group; CN: *Camellia nitidissima* group; ATP: *Akebia trifoliata* peels group; BE: bamboo leaf group; SML: silymarin (positive) group.

Alcohol metabolism is accompanied by the production of ROS, which further induce oxidative stress and antioxidant system disorders in the liver [38]. Reactive aldehydes, such as MDA, can be formed when lipid-free radicals are bound with oxygen molecules; thus, the MDA level reflects the status of oxidative stress [38]. As shown in Figure 5a, mice in the model group had higher levels of MDA in the liver than those in the control group (*p* < 0.01), hinting that oxidative stress occurred in the liver after acute exposure to alcohol. Mulberry leaf (ML), sweet tea (ST), vine tea (VT), *Akebia trifoliata* peel (ATP), and silymarin lowered the MDA level (all *p* < 0.05). In particular, ATP extract decreased the MDA level by 62.83% compared to the model group, which could be because it contains compounds such as chlorogenic acid, rutin, and ellagic acid [27]. This result indicated that ATP could effectively inhibit the oxidative stress induced by alcohol.

The activities of GSH-Px and SOD as well as the concentration of GSH in the liver were also analyzed. The levels of GSH, GSH-Px, and SOD in the model group were all reduced compared to the control group (all *p* < 0.05), showing that the hepatic antioxidant system was disrupted by alcohol. However, *Rabdosia serra* kudo (RSK), mulberry leaf (ML), sweet tea (ST), vine tea (VT), *Camellia nitidissima* (CN), bamboo leaf (BE), and silymarin increased the concentration of GSH in the liver (all *p* < 0.05), especially RSK and BE. As shown in Figure 5c, mulberry leaf (ML) and broadleaf holly leaf (BHL) extracts enhanced the activity of GSH-Px (*p* < 0.05 and *p* < 0.001, respectively), but *Akebia trifoliata* peel (ATP) extract inhibited the activity of GSH-Px. Moreover, silymarin increased the activity of SOD (*p* < 0.001), but broadleaf holly leaf (BHL) and *Akebia trifoliata* peel (ATP) extracts reduced the activity of SOD, and other tea-like plant extracts did not influence the activity of SOD (all *p* > 0.05). In addition, MDA, GSH, GSH-Px, and SOD are all in vivo antioxidant indicators, and these indicators might not be consistent, as reported in the literature [46,47].

Overall, alcohol induced lipid peroxidation and disrupted the antioxidant system in the livers of mice. However, ML, ST, VT, and ATP inhibited the lipid peroxidation induced by alcohol in the liver. Moreover, RSK, ML, ST, VT, CN, and BE enhanced the activities of antioxidant enzymes.

### 3.6. The Effects of Tea-like Plants on Inflammation in the Liver

**Figure 6:**
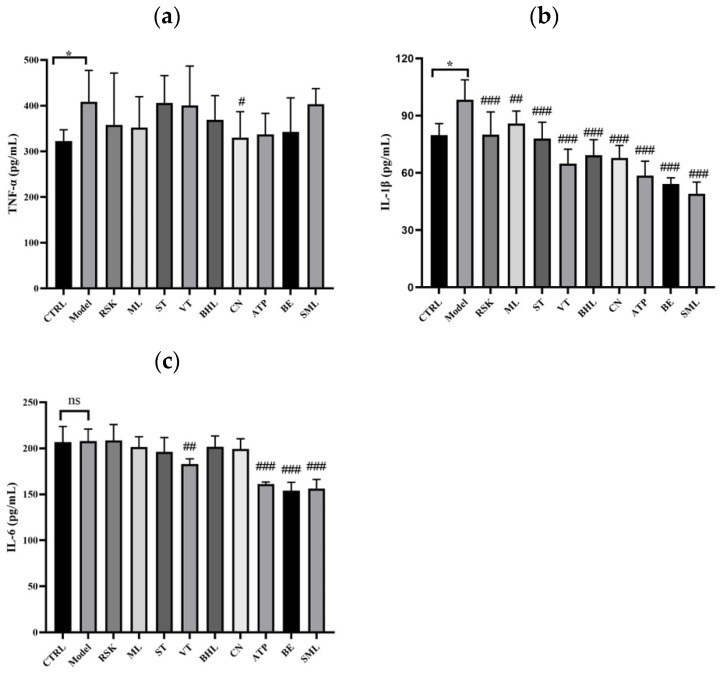
Effects of eight tea-like plants and silymarin on inflammation in the liver: (**a**) hepatic tumor necrosis factor-α (TNF-α); (**b**) hepatic interleukin (IL)-1β; (**c**) hepatic IL-6. * *p* < 0.05 in the comparison between control group and model group. “ns” means there were no significant differences between control group and model group. # *p* < 0.05, ## *p* < 0.01, and ### *p* < 0.001 in the comparison between intervention group and model group. CTRL: control group; Model: model group; RSK: *Rabdosia serra* kudo group; ML: mulberry leaf group; ST: sweet tea group; VT: vine tea group; BHL: broadleaf holly leaf group; CN: *Camellia nitidissima* group; ATP: *Akebia trifoliata* peels group; BE: bamboo leaf group; SML: silymarin (positive) group.

The results of TNF-α, IL-1β, and IL-6 in liver are shown Figure 6. Figure 6a,b shows that the levels of TNF-α and IL-1β were significantly elevated by alcohol (both *p* < 0.05), showing that acute alcohol exposure induced inflammation in the liver, whereas *Camellia nitidissima* (CN) extract lowered the hepatic level of TNF-α (*p* < 0.05). Additionally, all tea-like plant extracts and silymarin alleviated the increase in IL-1β induced by alcohol (all *p* < 0.01). As shown in Figure 6c, there were no significant differences in the level of IL-6 between the model group and control group, but vine tea (VT), *Akebia trifoliata* peel (ATP), and bamboo leaf (BE) extracts decreased the level of IL-6 (all *p* < 0.01).

Overall, eight tea-like plant extracts ameliorated the inflammation induced by alcohol in the liver, especially CN. The anti-inflammatory effects of CN were also reported in a previous study. Pre-treatment of CN for 7 days decreased the levels of TNF-α, IL-1β, and IL-6 in an acute liver injury model of CCl_4_ in a dose-dependent manner [41].

### 3.7. Effects of Tea-like Plants on Gut Microbiota Diversity and Composition

**Figure 7:**
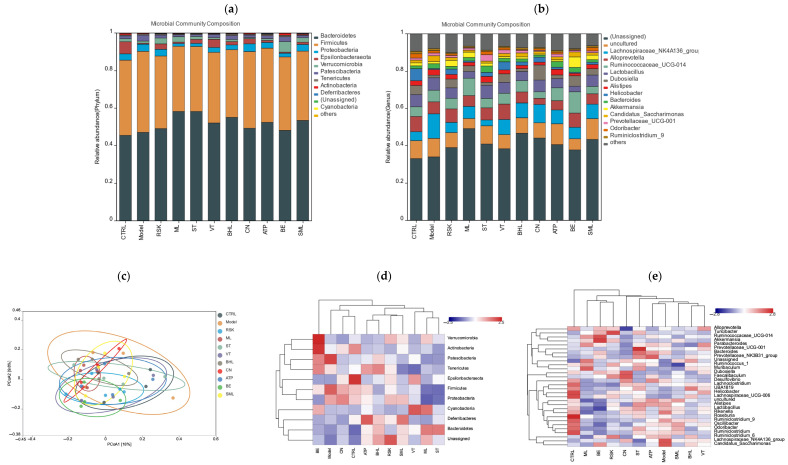
Effects of eight tea-like plants and silymarin on the composition of gut microbiota in alcohol-treated mice: (**a**) PCoA analysis; (**b**) relative abundance of gut microbiota at the phylum level among all groups; (**c**) relative abundance of gut microbiota at the genus level among all groups; (**d**) relative abundance of bacterial taxa at the phylum level based on heatmaps with cluster analysis; (**e**) relative abundance of bacterial taxa at the genus level based on heatmaps with cluster analysis. CTRL: control group; Model: model group; RSK: *Rabdosia serra* kudo group; ML: mulberry leaf group; ST: sweet tea group; VT: vine tea group; BHL: broadleaf holly leaf group; CN: *Camellia nitidissima* group; ATP: *Akebia trifoliata* peels group; BE: bamboo leaf group; SML: silymarin (positive) group.

Gut microbiota is closely connected with alcoholic liver disease [8]. In this study, ACE, Chao1, Simpson, and Shannon’s E indices were used to evaluate α diversity of gut microbiota. As shown in Table 2, there were no significant differences among any of the groups (all *p* > 0.05), indicating that alcohol and tea-like plant extracts did not affect the richness and diversity in gut microbiota. Moreover, β-diversity was also used to analyze the differences in composition and structure among all groups, and a closer distance means that the structure of gut microbiota is more similar. Based on PCoA analysis (shown in Figure 7c), alcohol exposure reshaped the structure of gut microbiota, compared with that of the control group. As shown in Figure 7d,e, species abundance cluster analysis found that alcohol exposure also changed the composition of gut microbiota at the phylum and genus levels. At the phylum level, the model group had high relative abundance of *Firmicutes* and *Patescibacteria*, while the control group had high relative abundance of *Actinobacteria* and *Epsilonbacteraeota*. Additionally, the *Camellia nitidissima* (CN) group was in the same cluster as the control group, indicating that the compositions of gut microbiota at the phylum level were similar between the CN group and control group.

**Figure 8:**
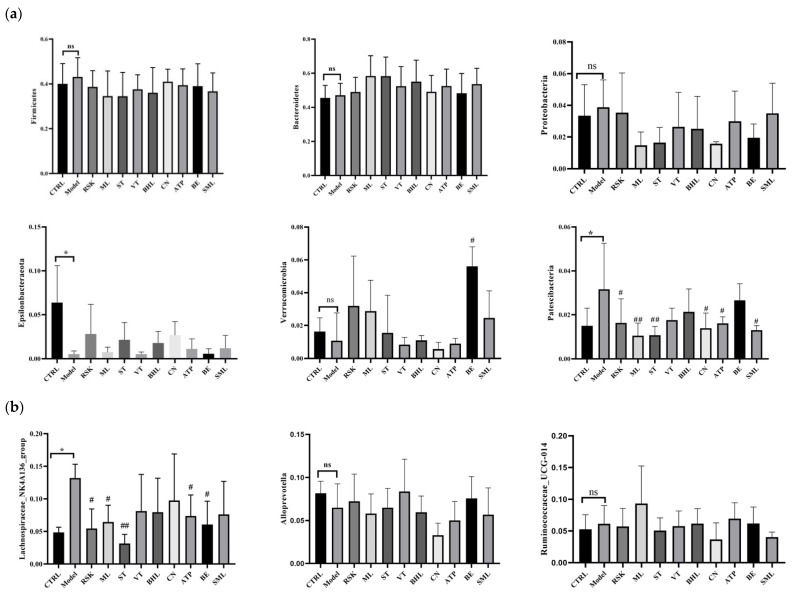

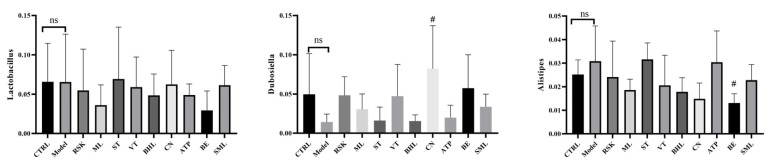
Effects of eight tea-like plants and silymarin on the composition of gut microbiota in alcohol-treated mice: (**a**) relative abundance of top 6 bacteria at phylum level; (**b**) relative abundance of top 6 bacteria at genus level. * *p* < 0.05 in the comparison between control group and model group. “ns” means there were no significant differences between control group and model group. # *p* < 0.05, and ## *p* < 0.01 in the comparison between intervention group and model group. CTRL: control group; Model: model group; RSK: *Rabdosia serra* kudo group; ML: mulberry leaf group; ST: sweet tea group; VT: vine tea group; BHL: broadleaf holly leaf group; CN: *Camellia nitidissima* group; ATP: *Akebia trifoliata* peels group; BE: bamboo leaf group; SML: silymarin (positive) group.

As shown in Figure 7a, the main bacteria at the phylum level of all groups were *Bacteroidetes*, *Firmicutes*, *Proteobacteria*, *Epsilonbacteraeota*, *Verrucomicrobia*, and *Patescibacteria*. Compared with the control group, the model group had a lower relative abundance of *Epsilonbacteraeota* but higher relative abundance of *Patescibacteria* (Figure 8a). A higher relative abundance of *Patescibacteria* induced by alcohol was also reported in other studies, and *Patescibacteria* is closely related to alcoholic liver disease [48,49]. *Rabdosia serra* kudo (RSK), mulberry leaf (ML), sweet tea (ST), *Camellia nitidissima* (CN), and *Akebia trifoliata* peel (ATP) significantly decreased the relative abundance of *Patescibacteria* (all *p* < 0.05). Among them, ST and ML decreased relative abundance of *Patescibacteria* even more than silymarin. These results showed that RSK, ML, ST, CN, and ATP regulated gut microbiota disorders caused by alcohol in order to ameliorate acute alcoholic liver injury. Additionally, bamboo leaf (BE) extract increased the abundance of *Verrucomicrobia* remarkably. Although there were no differences in *Bacteroidetes* or *Firmicutes* between the model group and control group, previous studies reported fewer *Bacteroidetes* but more *Firmicutes* in obesity [50,51]. The reason for these differences might be that 15-day alcohol exposure does not induce obesity; thus, differences in *Bacteroidetes* and *Firmicutes* cannot be seen in the comparison between model and control groups.

The changes in gut microbiota at the genus level were also analyzed and are shown in Figure 7b and Figure 8b. Among all groups, the richest bacteria at the genus level were *Lachnospiraceae*_NK4A136_group, *Alloprevotella*, *Ruminococcaceae*_UCG-014, *Lactobacillus*, *Dubosiella*, and *Alistipes*. The relative abundance of *Lachnospiraceae*_NK4A136_group was increased by alcohol (*p* < 0.05), while *Rabdosia serra* kudo (RSK), mulberry leaf (ML), sweet tea (ST), *Camellia nitidissima* (CN), *Akebia trifoliata* peel (ATP), and bamboo leaf (BE) extracts reduced its abundance (all *p* < 0.05). The higher abundance of *Lachnospiraceae*_NK4A136_group in mice exposed to alcohol was also reported in a previous study [52]. Another animal study showed the positive correlation between *Lachnospiraceae* and alcoholic liver injury [53]. *Lachnospiraceae* had a close association with inflammation. The abundance of *Lachnospiraceae* was positively associated with cyclooxygenase-2 (COX-2) and inducible nitric oxide synthase (iNOS), which further produced prostaglandin E2 (PGE2) and NO to induce inflammation [29]. Thus, RSK, ML, ST, CN, ATP, and BE probably ameliorated inflammation via regulating the abundance of *Lachnospiraceae*_NK4A136_group. Additionally, CN increased the abundance of *Dubosiella*, and BE decreased the abundance of *Alistipes*.

**Figure 9:**
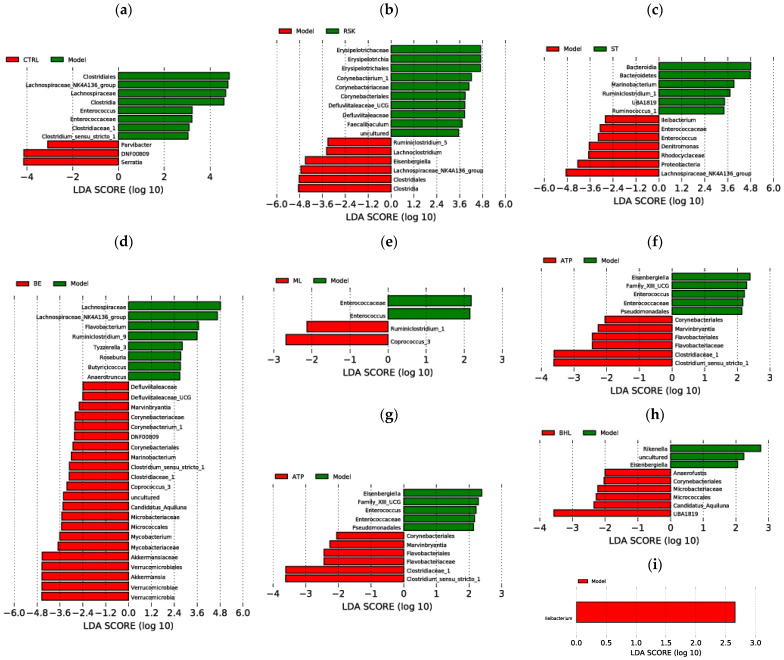
LEfSe analysis results: (**a**) CTRL vs. Model; (**b**) Model vs. RSK; (**c**) Model vs. ST; (**d**) Model vs. BE; (**e**) Model vs. ML; (**f**) Model vs. ATP; (**g**) Model vs. CN; (**h**) Model vs. BDL; (**i**) Model vs. VT. CTRL: control group; Model: model group; RSK: *Rabdosia serra* kudo group; ML: mulberry leaf group; ST: sweet tea group; VT: vine tea; BHL: broadleaf holly leaf group; CN: *Camellia nitidissima* group; ATP: *Akebia trifoliata* peels group; BE: bamboo leaf group.

LEfSe analysis was further used to identify unique bacteria from the phylum to genus level in each group, and LDA score threshold was set as 2. As shown in Figure 9a, the biomarkers of the model group were *Clostridiale* order, *Lachnospiraceae* family, *Enterococcaceae* family, *Clostridiaceae*_1 family, *Clostridia* class, *Enterococcus* genus, *Clostridium_sensu_stricto*_1 genus, and *Lachnospiraceae*_NK4A136_group genus, compared with the control group. Tea-like plant extracts reversed the gut microbiota changes induced by alcohol. Compared with the model group, *Rabdosia serra* kudo (RSK) inhibited the alcohol-induced increase in *Clostridiale* order, *Clostridia* class, and *Lachnospiraceae*_NK4A136_group genus. Sweet tea (ST) prevented the increase in *Enterococcaceae* family, *Enterococcus* genus, and *Lachnospiraceae*_NK4A136_group genus due to alcohol exposure and promoted the growth in *Bacteroidetes* genus and *Bacteroidia* class, compared to the model group. Bamboo leaf (BE) inhibited the increase in *Lachnospiraceae* family and *Lachnospiraceae*_NK4A136_group genus caused by alcohol. Moreover, mulberry leaf (ML), *Camellia nitidissima* (CN), and *Akebia trifoliata* peel (ATP) ameliorated the increase in *Enterococcaceae* family and *Enterococcus* genus caused by alcohol. Moreover, vine tea (VT) did not reverse the alcohol-induced changes in gut microbiota.

All in all, ST, ML, CN, and ATP had great regulatory action of gut microbiota and prevented changes in gut microbiota induced by alcohol.

### 3.8. Bioactive Compounds in Sweet Tea and Camellia nitidissima

**Figure 10:**
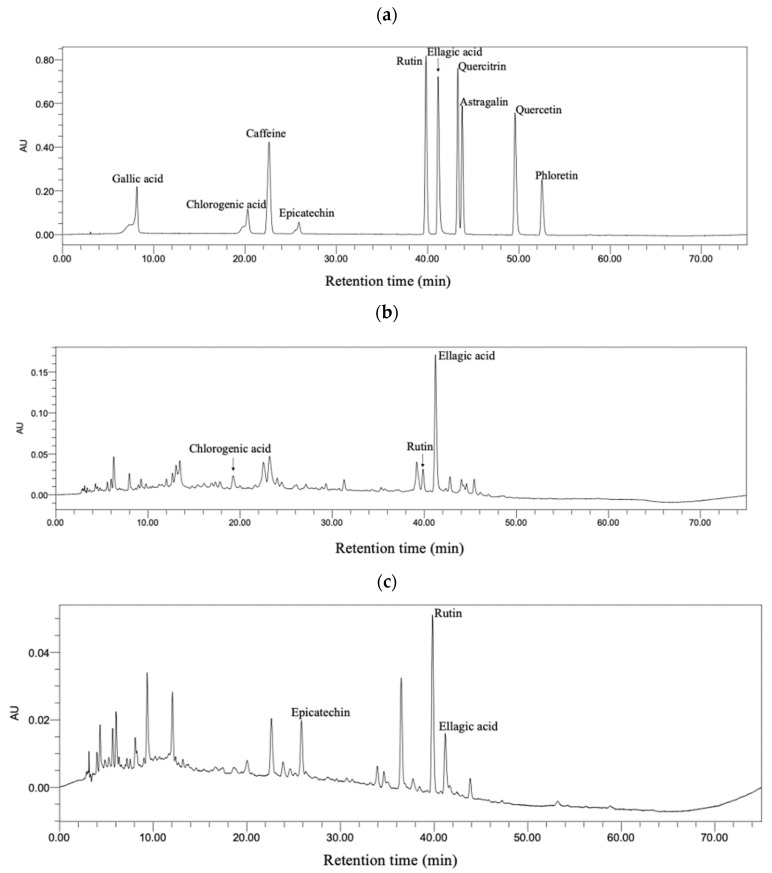
Chromatograms at 260 nm: (**a**) standards; (**b**) sweet tea; (**c**) *Camellia nitidissima*.

Comprehensively considering the above results, sweet tea and *Camellia nitidissima* were the two tea-like plants that had the greatest amelioration effect against acute alcoholic liver injury. Then, bioactive compounds of these two tea-like plant extracts were determined by HPLC. The chromatograms of standards, sweet tea, and *Camellia nitidissima* groups are shown in Figure 10. The bioactive compounds were identified by means of comparing the retention time and UV–visible spectra between standard and tea-like plant and quantified by peak area under maximum absorbance wavelength. The chromatograms of the standard compounds can be seen in Figure 10a. As shown in Figure 10b, chlorogenic acid, rutin, and ellagic acid were identified in the sweet tea extract, and their concentrations were 8.32 ± 0.84 mg/L, 19.19 ± 1.07 mg/L, and 39.70 ± 1.75 mg/L, respectively. As shown in Figure 10c, epicatechin, rutin, and ellagic acid were identified in the *Camellia nitidissima* extract, and their concentrations were 49.61 ± 6.35 mg/L, 13.89 ± 0.21 mg/L, and 9.31 ± 0.72 mg/L, respectively. A previous study reported that rutin reduced the levels of ALT and AST, increased the concentration of GSH, and decreased the level of TNF-α in alcohol-treated HepG2 cells [54]. Moreover, rutin activated ADH via H-bonds and van der Waals forces [55]. On the other hand, ellagic acid ameliorates oxidative stress, inflammation, and apoptosis in the livers of mice [56,57,58]. More importantly, ellagic acid also prevented the gut leakage and gut dysbiosis caused by alcohol [59]. Additionally, chlorogenic acid was reported to ameliorate alcoholic liver steatosis and fibrosis via decreasing ROS, maintaining the intestinal barrier, and alleviating gut dysbiosis [60,61]. Epicatechin alleviated oxidative stress, inflammation, and apoptosis in the liver in previous studies [62,63]. Thus, chlorogenic acid, epicatechin, rutin, and ellagic acid were probable to contribute a lot to the ameliorative effects of sweet tea and *Camellia nitidissima* on alcoholic liver injury.

## 4. Conclusions

In this study, the effects of eight tea-like plants on acute alcohol exposure were evaluated and compared. The results showed that sweet tea (ST), bamboo leaf (BE), mulberry leaf (ML), and *Camellia nitidissima* (CN) increased the activities of ADH or ALDH in the liver, while broadleaf holly leaf (BHL) inhibited the activity of ALDH. The majority of tea-like plant extracts except *Rabdosia serra* kudo (RSK) decreased levels of ALT or AST. Moreover, most of them, especially CN and ST, alleviated oxidative stress and inflammation in the liver. Furthermore, RSK, ML, ST, CN, and *Akebia trifoliata* peel (ATP) reversed the changes in gut microbiota induced by alcohol, such as decreased abundance of *Patescibacteria* (which was positively correlated with alcoholic liver disease) at the phylum level and the abundance of *Lachnospiraceae*_NK4A136_group at the genus level. All in all, the eight tea-like plants exerted hepatoprotective action against acute alcoholic liver injury to varying extents via enhancing the activities of alcoholic metabolism enzymes, ameliorating oxidative stress and inflammation in the liver, as well as regulating the gut microbiota (for example, decreasing the relative abundance of *Patescibacteria* at the phylum level), which could be drunk or developed into functional food for the prevention and management of alcoholic liver injury. Although various indicators were usually not consistent in this study, after comprehensive consideration of the results, sweet tea and *Camellia nitidissima* had the greatest hepatoprotective function against acute alcoholic liver injury. Chlorogenic acid, rutin, and ellagic acid were identified in sweet tea, and epicatechin, rutin, and ellagic acid were identified in *Camellia nitidissima*. These compounds could contribute to the hepatoprotective action of sweet tea and *Camellia nitidissima*.

## Figures and Tables

**Table 1:** The information of eight tea-like plants.

No.	Herbal Tea	Scientific Name	Place of Origin
1	Xi Huang Cao	*Rabdosia serra* (Maxim.) Kudo	Zhaoqing, Guangdong
2	Mulberry leaf	*Morus alba* Linn	Bozhou, Anhui
3	Sweet tea	*Rubus suavissimus* S. Lee	Jinxiu, Guangxi
4	Vine tea	*Ampelopsis grossedentata* (Hand.-Mazz.) W. T. Wang	Enshi, Hubei
5	Broadleaf holly leaf	*Ilex latifolia* Thunb.	Yuqing, Guizhou
6	Golden-flower tea	*Camellia nitidissima* C. W. Chi	Fangchenggang, Guangxi
7	Akebia pericarp	*Akebia trifoliata* (Thunb.) Koidz.	Haikou, Hainan
8	Bamboo leaf	*Lophatherum gracile* Brongn.	Chengdu, Sichuan

**Table 2:** α-diversity analysis of eight tea-like plants and silymarin on gut microbiota of alcohol-treated mice.

Group	ACE Index	Chao1 Index	Simpson Index	Shannon’s E Index
CTRL	1053.55 ± 24.94	8.63 ± 2.38	0.40 ± 0.05	1.13 ± 0.11
Model	1081.08 ± 27.69	5.88 ± 1.52	0.43 ± 0.02	1.01 ± 0.07
RSK	1081.00 ± 36.24	7.40 ± 2.22	0.41 ± 0.03	1.09 ± 0.08
ML	1091.98 ± 19.95	8.63 ± 4.08	0.49 ± 0.06	0.93 ± 0.12
ST	1072.50 ± 31.70	6.25 ± 0.83	0.49 ± 0.05	0.89 ± 0.08
VT	1033.98 ± 47.87	6.63 ± 2.41	0.44 ± 0.07	0.98 ± 0.15
BHL	1086.60 ± 21.53	8.88 ± 2.63	0.47 ± 0.04	0.98 ± 0.08
CN	1022.45 ± 65.62	8.91 ± 3.71	0.43 ± 0.04	1.04 ± 0.08
ATP	1097.35 ± 5.02	8.88 ± 2.77	0.45 ± 0.04	0.99 ± 0.12
BE	1054.68 ± 34.84	6.68 ± 1.26	0.41 ± 0.03	1.13 ± 0.09
SML	1070.85 ± 6.23	6.28 ± 1.06	0.44 ± 0.05	1.02 ± 0.13

CTRL: control group; Model: model group; RSK: *Rabdosia serra* kudo group; ML: mulberry leaf group; ST: sweet tea group; VT: vine tea group; BHL: broadleaf holly leaf group; CN: *Camellia nitidissima* group; ATP: *Akebia trifoliata* peels group; BE: bamboo leaf group; SML: silymarin (positive) group.

## Data Availability

The original contributions presented in the study are included in the article, further inquiries can be directed to the corresponding author.

## References

[1] Manthey J., Shield K.D., Rylett M., Hasan O.S.M., Probst C., Rehm J. (2019). Global alcohol exposure between 1990 and 2017 and forecasts until 2030: A modelling study. Lancet.

[2] Wu X.Q., Fan X.D., Miyata T., Kim A., Ross C.K.C.-D., Ray S., Huang E., Taiwo M., Arya R., Wu J.G. (2023). Recent advances in understanding of pathogenesis of alcohol-associated liver disease. Annu. Rev. Pathol.-Mech. Dis..

[3] Singal A.K., Mathurin P. (2021). Diagnosis and treatment of alcohol-associated liver disease a review. JAMA-J. Am. Med. Assoc..

[4] Huang D.Q., Mathurin P., Cortez-Pinto H., Loomba R. (2023). Global epidemiology of alcohol-associated cirrhosis and HCC: Trends, projections and risk factors. Nat. Rev. Gastroenterol. Hepatol..

[5] Addolorato G., Ponziani F.R., Dionisi T., Mosoni C., Vassallo G.A., Sestito L., Petito V., Picca A., Marzetti E., Tarli C. (2020). Gut microbiota compositional and functional fingerprint in patients with alcohol use disorder and alcohol-associated liver disease. Liver Int..

[6] Meng X., Li S., Li Y., Gan R.Y., Li H.B. (2018). Gut microbiota’s relationship with liver disease and role in hepatoprotection by dietary natural products and probiotics. Nutrients.

[7] Dubinkina V.B., Tyakht A.V., Odintsova V.Y., Yarygin K.S., Kovarsky B.A., Pavlenko A.V., Ischenko D.S., Popenko A.S., Alexeev D.G., Taraskina A.Y. (2017). Links of gut microbiota composition with alcohol dependence syndrome and alcoholic liver disease. Microbiome.

[8] Zhu L., Wang Y.X., Pan C.Q., Xing H.C. (2023). Gut microbiota in alcohol-related liver disease: Pathophysiology and gut-brain cross talk. Front. Pharmacol..

[9] Long P., Cui Z.H., Wang Y.L., Zhang C.H., Zhang N., Li M.H., Xiao P.G. (2014). Commercialized non-*Camellia* tea: Traditional function and molecular identification. Acta Pharm. Sin. B.

[10] Liu H., Zhang R.G., Zhou B.F., Shen Z., Chen X.Y., Gao J., Wang B.S. (2023). Chromosome-scale genome assembly of sweet tea (*Lithocarpus polystachyus* Rehder). Sci. Data.

[11] Saimaiti A., Huang S.Y., Xiong R.G., Wu S.X., Zhou D.D., Yang Z.J., Luo M., Gan R.Y., Li H.B. (2022). Antioxidant capacities and polyphenol contents of kombucha beverages based on vine tea and sweet tea. Antioxidants.

[12] Zhang Q.L., Zhao Y.F., Zhang M.Y., Zhang Y.L., Ji H.F., Shen L. (2021). Recent advances in research on vine tea, a potential and functional herbal tea with dihydromyricetin and myricetin as major bioactive compounds. J. Pharm. Anal..

[13] Lin L.Z., Zhao H.F., Dong Y., Yang B., Zhao M.M. (2012). Macroporous resin purification behavior of phenolics and rosmarinic acid from *Rabdosia serra* (MAXIM.) HARA leaf. Food Chem..

[14] Liu G.L., Xu W., Liu X.J., Yan X.L., Chen J. (2020). Two new abietane diterpenoids from the leaves of *Rabdosia serra*. J. Asian Nat. Prod. Res..

[15] Wüpper S., Lüersen K., Rimbach G. (2020). Chemical composition, bioactivity and safety aspects of kuding tea-from beverage to herbal extract. Nutrients.

[16] Wu H.L., Chen Y.L., Yu Y.Y., Zang J., Wu Y.K., He Z. (2017). *Ilex latifolia* Thunb protects mice from HFD-induced body weight gain. Sci. Rep..

[17] Chen G.J., Xie M.H., Dai Z.Q., Wan P., Ye H., Zeng X.X., Sun Y. (2018). Kudingcha and Fuzhuan Brick Tea Prevent Obesity and Modulate Gut Microbiota in High-Fat Diet Fed Mice. Mol. Nutr. Food Res..

[18] Cui W.Y., Luo K.Y., Xiao Q., Sun Z.Y., Wang Y.F., Cui C.F., Chen F.C., Xu B., Shen W.J., Wan F.C. (2023). Effect of mulberry leaf or mulberry leaf extract on glycemic traits: A systematic review and meta-analysis. Food Funct..

[19] Ma G.Q., Chai X.Y., Hou G.G., Zhao F.L., Meng Q.G. (2022). Phytochemistry, bioactivities and future prospects of mulberry leaves: A review. Food Chem..

[20] Xiong R.G., Wu S.X., Cheng J., Saimaiti A., Liu Q., Shang A., Zhou D.D., Huang S.Y., Gan R.Y., Li H.B. (2023). Antioxidant activities, phenolic compounds, and sensory acceptability of kombucha-fermented beverages from bamboo leaf and mulberry leaf. Antioxidants.

[21] Cheng Y.Q., Wan S.Q., Yao L.N., Lin D., Wu T., Chen Y.J., Zhang A.L., Lu C.F. (2023). Bamboo leaf: A review of traditional medicinal property, phytochemistry, pharmacology, and purification technology. J. Ethnopharmacol..

[22] Koide C.L.K., Collier A.C., Berry M.J., Panee J. (2011). The effect of bamboo extract on hepatic biotransforming enzymes—Findings from an obese-diabetic mouse model. J. Ethnopharmacol..

[23] Ge L., Lin B.H., Mo J.G., Chen Q.H., Su L., Li Y.J., Yang K.D. (2019). Composition and antioxidant and antibacterial activities of essential oils from three yellow *Camellia* species. Trees-Struct. Funct..

[24] He D.Y., Li X.Y., Sai X., Wang L.L., Li S.Y., Xu Y.P. (2018). *Camellia nitidissima* CW Chi: A review of botany, chemistry, and pharmacology. Phytochem. Rev..

[25] Wang Y.Q., Peng X., Tang Q., Yu D.Y., Luo Y.Y., Shi L.Y., Huang L.D., Mou H.M., Tang L., Feng B.M. (2009). Active fraction of IgE-mediated type I allergy from Camellia nitidissima. Cent. South Pharm..

[26] Wu S.X., Xiong R.G., Cheng J., Xu X.Y., Tang G.Y., Huang S.Y., Zhou D.D., Saimaiti A., Gan R.Y., Li H.B. (2023). Preparation, antioxidant activities and bioactive components of kombucha beverages from golden-flower tea (*Camellia petelotii*) and honeysuckle-flower tea (*Lonicera japonica*). Foods.

[27] Luo M., Zhou D.D., Shang A., Gan R.Y., Li H.B. (2021). Influences of microwave-assisted extraction parameters on antioxidant activity of the extract from *Akebia trifoliata* peels. Foods.

[28] Wang J., Ren H., Xu Q.L., Zhou Z.Y., Wu P., Wei X.Y., Cao Y., Chen X.X., Tan J.W. (2015). Antibacterial oleanane-type triterpenoids from pericarps of *Akebia trifoliata*. Food Chem..

[29] Wang X.Y., Yu N.X., Wang Z.L., Qiu T.T., Jiang L., Zhu X.M., Sun Y., Xiong H. (2020). *Akebia trifoliata* pericarp extract ameliorates inflammation through NF-κB/MAPK signaling pathways and modifies gut microbiota. Food Funct..

[30] Afifi N.A., Ibrahim M.A., Galal M.K. (2018). Hepatoprotective influence of quercetin and ellagic acid on thioacetamide-induced hepatotoxicity in rats. Can. J. Physiol. Pharmacol..

[31] Lee J.H., Won J.H., Choi J.M., Cha H.H., Jang Y.J., Park S., Kim H.G., Kim H.C.U., Kim D.K. (2014). Protective effect of ellagic acid on concanavalin A-induced hepatitis via toll-like receptor and mitogen-activated protein kinase/nuclear factor kb signaling pathways. J. Agric. Food Chem..

[32] Srigopalram S., Jayraaj I.A., Kaleeswaran B., Balamurugan K., Ranjithkumar M., Kumar T.S., Park J.I., Nou I.S. (2014). Ellagic acid normalizes mitochondrial outer membrane permeabilization and attenuates inflamma-tion-mediated cell proliferation in experimental liver cancer. Appl. Biochem. Biotechnol..

[33] Zhang C., Hu J.J., Sheng L., Yuan M., Wu Y., Chen L., Wang G.H., Qiu Z.P. (2019). Ellagic acid ameliorates AKT-driven hepatic steatosis in mice by suppressing *de novo* lipogenesis via the AKT/SREBP-1/FASN pathway. Food Funct..

[34] Meng X., Li Y., Li S., Gan R.Y., Li H.B. (2018). Natural Products for Prevention and Treatment of Chemical-Induced Liver Injuries. Compr. Rev. Food Sci. Food Saf..

[35] Wang F., Zhang Y.J., Zhou Y., Li Y., Zhou T., Zheng J., Zhang J.J., Li S., Xu D.P., Li H.B. (2016). Effects of Beverages on Alcohol Metabolism: Potential Health Benefits and Harmful Impacts. Int. J. Mol. Sci..

[36] Li B.Y., Mao Q.Q., Zhou D.D., Luo M., Gan R.Y., Li H.Y., Huang S.Y., Saimaiti A., Shang A., Li H.B. (2021). Effects of tea against alcoholic fatty liver disease by modulating gut microbiota in chronic alcohol-exposed mice. Foods.

[37] Cao S.Y., Li B.Y., Gan R.Y., Mao Q.Q., Wang Y.F., Shang A., Meng J.M., Xu X.Y., Wei X.L., Li H.B. (2020). The in vivo antioxidant and hepatoprotective actions of selected Chinese teas. Foods.

[38] Jiang Y.C., Zhang T., Kusumanchi P., Han S., Yang Z.H., Liangpunsakul S. (2020). Alcohol metabolizing enzymes, microsomal ethanol oxidizing system, cytochrome P450 2E1, catalase, and aldehyde dehydrogenase in alcohol-associated liver disease. Biomedicines.

[39] Tran S., Nowicki M., Chatterjee D., Gerlai R. (2015). Acute and chronic ethanol exposure differentially alters alcohol dehydrogenase and aldehyde dehydrogenase activity in the zebrafish liver. Prog. Neuro-Psychopharmacol. Biol. Psychiatry.

[40] Pohl K., Moodley P., Dhanda A.D. (2021). Alcohol’s impact on the gut and liver. Nutrients.

[41] Zhang X.M., Feng J., Su S.F., Huang L. (2020). Hepatoprotective effects of *Camellia nitidissima* aqueous ethanol extract against CCl4-induced acute liver injury in SD rats related to Nrf2 and NF-κB signalling. Pharm. Biol..

[42] Liu X.K., Wang K.Y., Cai G.Z., Li H.T., Guo Y.L., Gong J.Y. (2023). Comparative chemical diversity and antioxidant activities of three species of *Akebia* herbal medicines. Arab. J. Chem..

[43] Wang X.Y., Yu N.X., Peng H.L., Hu Z.Y., Sun Y., Zhu X.M., Jiang L., Xiong H. (2019). The profiling of bioactives in *Akebia trifoliata* pericarp and metabolites, bioavailability and in vivo anti-inflammatory activities in DSS-induced colitis mice. Food Funct..

[44] Jeon S., Carr R. (2020). Alcohol effects on hepatic lipid metabolism. J. Lipid Res..

[45] Oku H., Ogawa Y., Iwaoka E., Yamaguchi Y., Kagota S., Kazumasa S., Kunitomo M., Ishiguro K. (2011). Preventive effects of the extract of kinka-cha, a folk tea, on a rat model of metabolic syndrome. J. Nat. Med..

[46] Li B.Y., Li H.Y., Zhou D.D., Huang S.Y., Luo M., Gan R.Y., Mao Q.Q., Saimaiti A., Shang A., Li H.B. (2021). Effects of Different Green Tea Extracts on Chronic Alcohol Induced-Fatty Liver Disease by Ameliorating Oxidative Stress and Inflammation in Mice. Oxidative Med. Cell. Longev..

[47] Munkong N., Somnuk S., Jantarach N., Ruxsanawet K., Nuntaboon P., Kanjoo V., Yoysungnoen B. (2023). Red Rice Bran Extract Alleviates High-Fat Diet-Induced Non-Alcoholic Fatty Liver Disease and Dyslipidemia in Mice. Nutrients.

[48] Ran B.B., Guo C.E., Li W.D., Li W.S., Wang Q., Qian J.X., Li H.L. (2021). Sea buckthorn (*Hippophae rhamnoides* L.) fermentation liquid protects against alcoholic liver disease linked to regulation of liver metabolome and the abundance of gut microbiota. J. Sci. Food Agric..

[49] Li H.L., Xie Z.Y., Zhang Y., Liu Y., Niu A.J., Liu Y.Y., Zhang L.B., Guan L.L. (2021). *Rosa rugosa* polysaccharide attenuates alcoholic liver disease in mice through the gut-liver axis. Food Biosci..

[50] Ley R.E., Turnbaugh P.J., Klein S., Gordon J.I. (2006). Microbial ecology—Human gut microbes associated with obesity. Nature.

[51] Turnbaugh P.J., Ley R.E., Mahowald M.A., Magrini V., Mardis E.R., Gordon J.I. (2006). An obesity-associated gut microbiome with increased capacity for energy harvest. Nature.

[52] Yi Z.W., Liu X.F., Liang L.H., Wang G.Q., Xiong Z.Q., Zhang H., Song X., Ai L.Z., Xia Y.J. (2021). Antrodin A from *Antrodia camphorata* modulates the gut microbiome and liver metabolome in mice exposed to acute alcohol intake. Food Funct..

[53] Tian X.Z., Li R., Jiang Y.M., Zhao F., Yu Z.S., Wang Y.Q., Dong Z.X., Liu P., Li X.K. (2020). *Bifidobacterium breve* ATCC15700 pretreatment prevents alcoholic liver disease through modulating gut microbiota in mice exposed to chronic alcohol intake. J. Funct. Food..

[54] Lee S., Lee J., Lee H., Sung J. (2019). Relative protective activities of quercetin, quercetin-3-glucoside, and rutin in alcohol-induced liver injury. J. Food Biochem..

[55] Huang X.J., Zhang S.Y., Li Y.S., Yang X., Li N., Zeng G.F., Chen F.P., Tuo X. (2022). Insight into the binding characteristics of rutin and alcohol dehydrogenase: Based on the biochemical method, spectroscopic experimental and molecular model. J. Photochem. Photobiol. B-Biol..

[56] Aishwarya V., Solaipriya S., Sivaramakrishnan V. (2021). Role of ellagic acid for the prevention and treatment of liver diseases. Phytother. Res..

[57] Zhao L., Mehmood A., Soliman M.M., Iftikhar A., Iftikhar M., Aboelenin S.M., Wang C.T. (2021). Protective effects of ellagic acid against alcoholic liver disease in mice. Front. Nutr..

[58] Siroma T.K., Machate D.J., Zorgetto-Pinheiro V.A., Figueiredo P.S., Marcelino G., Hiane P.A., Bogo D., Pott A., Cury E.R.J., Guimaraes R.D.A. (2022). Polyphenols and ω-3 pUFAs: Beneficial outcomes to obesity and its related metabolic diseases. Front. Nutr..

[59] Kim D.H., Sim Y., Hwang J.H., Kwun I.S., Lim J.H., Kim J., Kim J.I., Baek M.C., Akbar M., Seo W. (2021). Ellagic acid prevents binge alcohol-induced leaky gut and liver injury through inhibiting gut dysbiosis and oxidative stress. Antioxidants.

[60] Kim H., Pan J.H., Kim S.H., Lee J.H., Park J.W. (2018). Chlorogenic acid ameliorates alcohol-induced liver injuries through scavenging reactive oxygen species. Biochimie.

[61] Zhu H.K., Jiang W.H., Liu C., Wang C., Hu B., Guo Y.H., Cheng Y.L., Qian H. (2022). Ameliorative effects of chlorogenic acid on alcoholic liver injury in mice via gut microbiota informatics. Eur. J. Pharmacol..

[62] Sinha M., Das D.K., Manna K., Datta S., Ray T., Sil A.K., Dey S. (2012). Epicatechin ameliorates ionising radiation-induced oxidative stress in mouse liver. Free Radic. Res..

[63] Wu H., Xie Y.N., Xu Y.L., Hu Z.H., Wan X., Huang H.C., Huang D.B. (2020). Protective effect of epicatechin on APAP-induced acute liver injury of mice through anti-inflammation and apoptosis inhibition. Nat. Prod. Res..

